# More tropical cyclones are striking coasts with major intensities at landfall

**DOI:** 10.1038/s41598-022-09287-6

**Published:** 2022-03-28

**Authors:** S. Wang, R. Toumi

**Affiliations:** Department of Physics, Imperial College London, London, SW7 2AZ UK

**Keywords:** Atmospheric science, Climate change

## Abstract

In this study, we show that the number of annual global tropical cyclone (TC) landfalls with major landfall intensity (LI ≥ 50 m s^−1^) has nearly doubled from 1982 to 2020. The lifetime maximum intensity (LMI) of global major landfalling TCs has been increasing by 0.8 m s^−1^ per decade (*p* < 0.05), but this significance of intensity change disappears at landfall (0.3 m s^−1^ per decade, *p* = 0.69). The lack of a significant LI trend is caused by the much larger variance of LI than that of LMI in all basins and explains why a significant count change of TCs with major intensity at landfall has only now emerged. Basin-wide TC trends of intensity and spatial distribution have been reported, but this long-term major TC landfall count change may be the most socio-economic significant.

## Introduction

Tropical cyclones (TCs) are one of the major global natural hazards. The landfall intensity (LI) dominates its destructive potential in coastal regions^1,2^. The TC intensity is conventionally measured by the maximum sustained surface wind and is categorised as a major TC when its sustained maximum is more than 50 m s^−1^ (Saffir–Simpson categories 3–5). Thermodynamic theories of an upper intensity limit^3,4^ and its environmental controls^5,6^ have advanced TC intensity understanding and prediction. The lifetime maximum intensity (LMI) of a TC is the intensity closest to its theoretical upper limit and has received much more attention than the intensity in any other period of its lifecycle. However, the location of LMI is usually hundreds of kilometres away from land^7^, and an intensity decay from the LMI to LI frequently occur before landfall^8^. The long-term TC socio-economic impact is ultimately driven by the number of intense storms making landfall.

Major TCs at landfall (LI ≥ 50 m s^−1^) are of particular interest as they account for most of the TC-related damage^9^. To date, no firm evidence has been found for a globally significant trend of the TC landfall intensity or frequency above an intensity threshold^10,11^. However, over oceans the fraction of major TCs and their LMI have both increased^12,13^. The global LMI location is also migrating towards the coasts^7^. Why have the landfall changes of TCs been so small making them difficult to detect?

Here we present a new analysis of global TC landfall changes. The major landfall count and the intensity change of major TCs are the foci of this study. For clarity the following terms are used in the rest of the study, unless otherwise indicated: “landfall” is for major landfall events with LIs of at least major intensity (i.e., LI ≥ 50 m s^−1^) and “TC” is for landfalling cyclones with LMIs of at least major intensity (i.e., LMI ≥ 50 m s^−1^). We will show that the annual global number of landfalls has increased significantly from 1982 to 2020.

## Results

**Figure 1:**
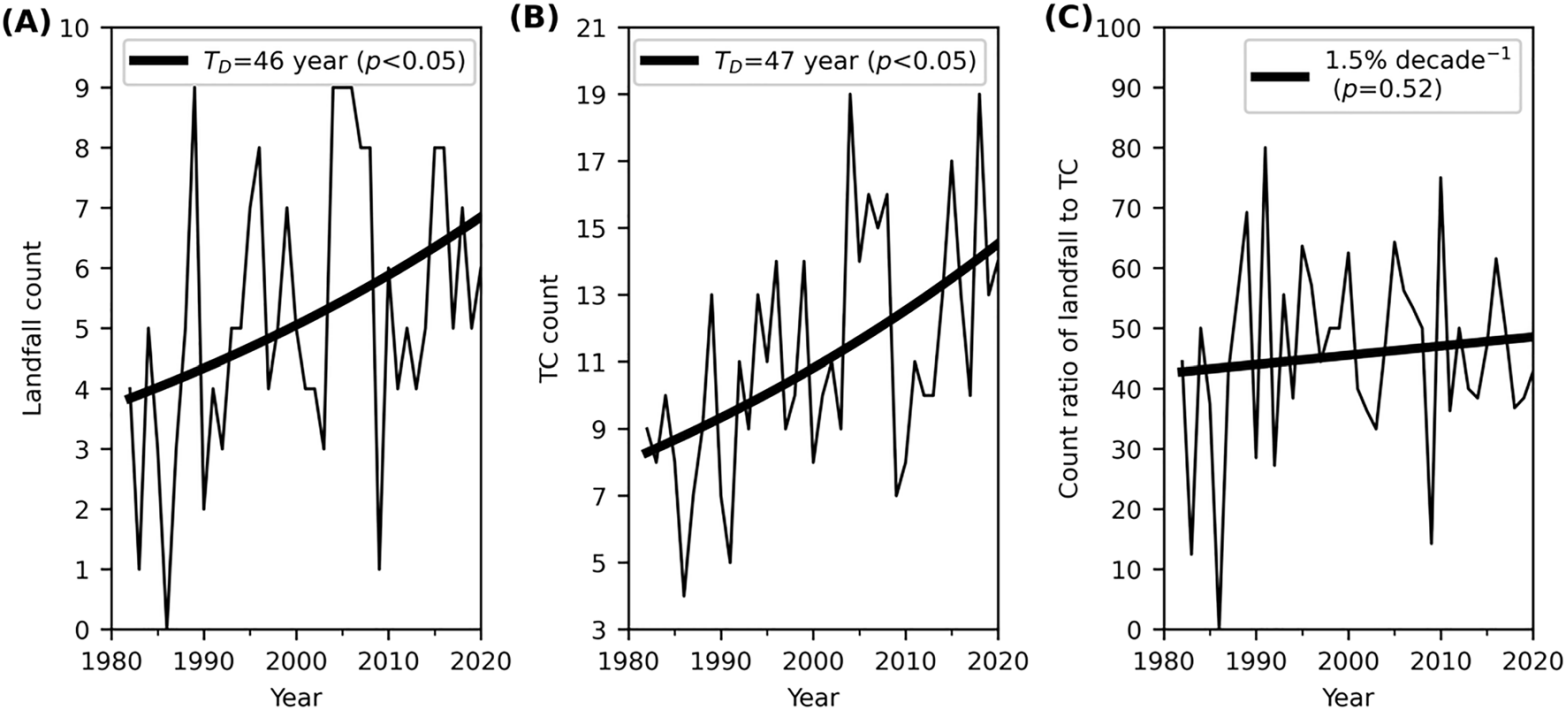
Observed annual (**a**) landfall count, (**b**) TC count, and (**c**) their count ratio. As defined in “Methods”, “landfall” and “TC” here are referred to as major landfall events with LIs of at least major intensity (≥ 50 m s^−1^), and landfalling storms with LMIs of at least major intensity, respectively. The thick line in (**a**,**b**) shows the Poisson regression fit. The fitted slope b_1_ in Eq. (1) is 0.015 ± 0.012 in (**a**) and 0.015 ± 0.008 in (**b**), respectively. The doubling time (T_D_) estimated by the Poisson regression is given in the legend. The thick line in (**c**) shows the linear trend of the count ratio that is not statistically significant.

Figure 1a shows an increase in the global number of landfalls with a doubling time (see “Methods”) of 46 years (*p* < 0.05). There has been an approximately doubling of landfalls from about four to seven annually over the last 40 years. However, this significant increase of landfalls is only observed at the global scale (Table 1). There is also an increase in the global number of TCs (Fig. 1b), showing a consistent and significant doubling time of 47 years (*p* < 0.05). A longer but still significant doubling of 56 years is also observed in the West Pacific (WPAC, Table 1). The significant increase of TCs and landfalls in Fig. 1 is not sensitive to the choice of the regression model (see “Methods”). Linear trend analysis also shows a significant increase in the annual number of landfalls (Fig. 1a, + 0.8 count per decade, *p* < 0.05) and TCs (Fig. 1b, + 1.6 count per decade, *p* < 0.05). Figure 1c shows that about 45% of TCs maintains the major intensity to land, and this fraction has not changed significantly (*p* = 0.52) for 1982–2020. All the significant changes shown in Fig. 1 remain for the period 1970–2020 (Fig. S1).

**Table 1:** Doubling time of landfall and TC counts, linear trends of annual mean TC LMI and LI, and one standard deviation (σ) of LMI and LI based on individual TCs.

	Global	WPAC	EPAC	NATL	NIO	SIO	SPAC
Landfall doubling time (year)	**46**	46	17	25	88	205	70
TC doubling time (year)	**47**	**56**	117	31	21	42	130
TC LMI trend (m s^−1^ per decade)	**0.8**	1.1	2.5	1.3	− 0.9	− 0.3	-0.4
TC LI trend (m s^−1^ per decade)	0.3	0.7	1.2	0.8	− 5.7	− 2.2	1.9
TC LMI σ (m s^−1^)	17	17	17	18	15	14	15
TC LI σ (m s^−1^)	30	29	33	22	32	30	28

Globally and for each region (WPAC, West Pacific; EPAC, East Pacific; NATL, North Atlantic; NIO, North Indian Ocean; SIO, South Indian Ocean; SPAC, South Pacific). Statistically significant (*p* < 0.05) doubling time and trend are shown in bold. As defined in “Methods”, “landfall” and “TC” here are referred to as major landfall events with LIs of at least major intensity (≥ 50 m s^−1^), and landfalling storms with LMIs of at least major intensity, respectively.

**Figure 2:**
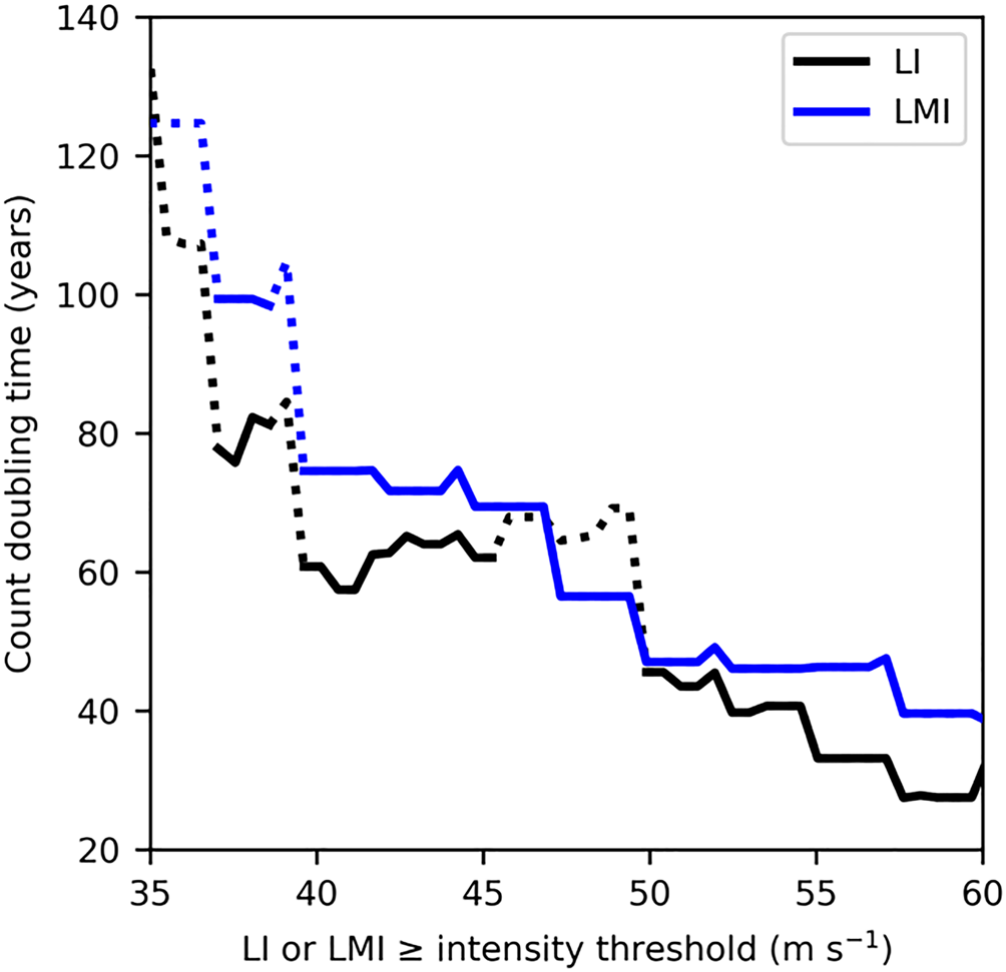
Sensitivity of count analysis. Global annual count doubling time from a Poisson regression of all the selected storms (LMI ≥ 33 m s^−1^) with LI (black) or LMI (blue) above different intensity thresholds. The significant doubling times (*p* < 0.05) are highlighted with the solid line. All the regressions are conducted with at least 30 years of non-zero observations.

The significant increases of TC and landfall counts in Fig. 1 are calculated based on the wind threshold of 50 m s^−1^ to define a major intensity. It is important to examine the sensitivity of these statistical significance to the choice of intensity thresholds. Figure 2 shows that the significant long-term changes can still be detected for intensity thresholds above 50 m s^−1^ with Poisson and linear (not shown) regressions. We do not find any significant poleward migration trend for either the annual mean latitude of landfalling LMI or landfall locations (Fig. S2). In our analysis we consider storm activities within 40° N/S, but we also find count increase of annual landfalls beyond 40° N/S, particularly for hurricanes making landfall in the US and Europe (Fig. S3).

**Figure 3:**
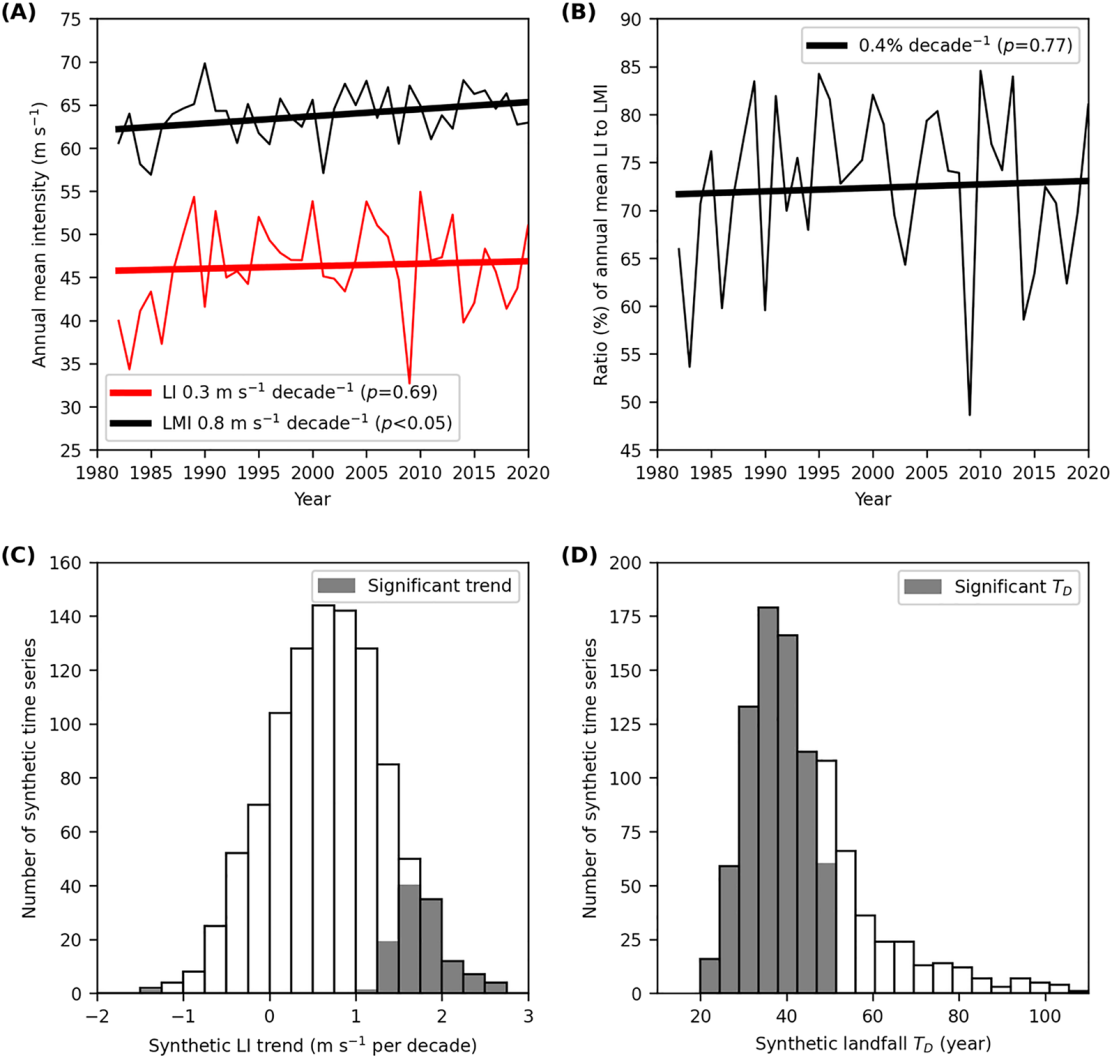
TC intensity statistics and simulations. (**a**) Annual mean trend of TC LI and LMI. (**b**) Trend of the ratio of annual mean TC LI to LMI. (**c**) Annual mean trends of synthetic TC LI. (**d**) Doubling time (T_D_) of synthetic annual landfalls. The dark bars in (**c**,**d**) highlight the significant synthetic LI trends and T_D_ (*p* < 0.05). As defined in “Methods”, “landfall” and “TC” here are referred to as major landfall events with LIs of at least major intensity (≥ 50 m s^−1^), and landfalling storms with LMIs of at least major intensity, respectively.

Not only is there a TC frequency increase, but the TC LMI has also increased by + 0.8 m s^−1^ per decade globally (*p* < 0.05, Fig. 3a), even though this trend is not significant in any of the individual basins (Table 1). This global LMI increase has also not been directly translated to detectable LI increase either globally or in any basin. The LI linear trend (+ 0.3 m s^−1^ per decade, Fig. 3a) is less than half of the LMI trend (+ 0.8 m s^−1^ per decade, Fig. 3a) and has not changed significantly (*p* = 0.69). Figure 3b shows that the LI trend can be partly understood as proportional to the LMI trend with a stabilized ratio (annual mean LI relative to LMI) of about 0.7.

If the TC LI were simply a stabilised fraction of TC LMI, from a statistical perspective, the LI trend might inherit the trend significance from LMI. A more important reason for the lack of significance for the TC LI trend in Fig. 3a is that not only is the trend signal smaller, but the noise is also larger. For example, the TC LI range from the 5th to 95th percentiles of 54 m s^−1^ (69–15 m s^−1^) is almost doubled relative to the range of TC LMI (29 m s^−1^; 80–51 m s^−1^). The standard deviation of LI is also larger than that of LMI globally and by regions (Table 1). Therefore, the mean reduction of LI from LMI and the much larger variance of LI both lead to a weaker and not significant LI trend.

To further illustrate this point, we simulate LI trends and landfall count changes (Fig. 3c,d). We generate 1000 synthetic LI time series for 1982–2020 by multiplying the observed LMI of each TC by a reduction factor (LI/LMI ratio) randomly sampled from the observed distribution of the factor. The mean and standard deviation of the synthetic LI trends are both 0.6 m s^−1^ per decade and the observed mean trend (Fig. 3a) is within the simulated range. The mean synthetic landfall doubling time is 45 h (Fig. 3d), which is in excellent agreement with the observation (Fig. 1a). About 90% of the synthetic LI trends are not significant (*p* < 0.05, Fig. 3c). However, 75% of the synthetic landfall doubling times are statistically significant (Fig. 3d). The annual mean reduction factor has been stationary (Fig. 3b), and LI values are highly variable (Table 1). Thus, the simulations show that it is more likely than not that the increase in LMI has increased the number of landfalls as major TCs, while their mean intensity at landfall has not changed.

Further analysis also reveals a weak relationship between global annual mean LMI and LI of all the selected landfalling storms (LMI ≥ 33 m s^−1^, *r* = 0.23, *p* = 0.16). Significant decadal correlation is found only when applying a Lanczos filter^14^ with 9-year low-pass filtering (*r* = 0.35, *p* < 0.05), but not for a box linear 9-year running mean filter (*r* = 0.32, *p* = 0.09). Considering the small sample size in individual basins, we calculated the mean LMI and LI changes of all the selected landfalling storms between two epochs (2001–2020 minus 1982–2000). The epochal analysis confirms the weak relationship between LI and LMI changes in all the basins (Table S1). The epochal absolute change of the regional mean LI is less than that of LMI in the West Pacific, East Pacific and North Atlantic, which account for 75% of global landfall counts. The epochal changes of LI and LMI even have opposite signs in the Indian Ocean basins. The South Pacific is the only basin with a significant positive epochal LI change. The decoupling of LI and LMI of all major and minor storms with landfalls in Table S1 echoes the much larger variation of TC LI compared to that of TC LMI shown in Table 1.

## Discussion and conclusions

The long-term change of landfalls was previously analyzed by Ref.^10^ for 1970–2010 and extended to 2017 in Ref.^11^. Both studies^10,11^ found that the global linear trend was close to be significant, e.g., with a *p* value of 0.06 for 1970–2010. Their conclusions were based on a finer land mask (see “Methods”) and conventional linear trend test. The counts are small, and this makes it difficult to assume Gaussian errors and determine significance levels for simple linear regression^15^. A Poisson process is a preferred tool for landfall events^16–21^. By fitting the landfall counts reported by Ref.^10^ (their Fig. 2a) and Ref.^11^ (their Fig. 1i) with a Poisson regression, we find a doubling time of 48 years (*p* = 0.02) for 1970–2010 and 70 years (*p* = 0.03) for 1970–2017. This is consistent with the significant landfall increase in our analysis for 1982–2020 and 1970–2020 with a courser land mask. The significant increase of global landfalls based on a Poisson regression is therefore not sensitive to the period examined or how landfall is defined. There is still a lack of significant trend by basin for the period 1970–2020, which is in line with Ref.^10^ and may be due to a large inter-basin variability, e.g., the US major landfall “drought” for 2006–2016. With the current analysis it is difficult to answer the question: is the increase of landfall counts associated with anthropogenic climate change or due to multidecadal internal variability of the climate system? The synthetic TC tracks downscaled from global climate analyses back to the nineteenth century can be useful to answer this question in future analysis^21^.

The LI trend can be partly understood as proportional to the LMI trend with a stabilized ratio. Since the LMI is expected to increase in a warming climate^22^ it is plausible that the LI should also proportionally increase with LMI and lead to an increase of landfall number. This is supported by our simulations of the landfall number trends.

However, compared to the LMI trend, a reduced mean trend of LI makes it more challenging to be detected. The detection problem is enhanced because of the much larger variability of LI compared to that of LMI. This larger variability of LI may be caused by a combined effect of multiple internal and environmental factors, for example, the progressive self-weakening process post LMI^23,24^, variable decay distance and translation speed from LMI to LI^7,25^, and changes in vertical wind shear and thermodynamic conditions when a TC approaches coastlines^26,27^. All the factors discussed above may be the reasons why this change has been difficult to detect. There is no compelling need to invoke a physical explanation of changes in the landfall process. The synthetic simulations show that the trends can be understood simply as a reflection of the previously reported increases in LMI and the inherent large (but stationary) variability of the decay to landfall.

We report, for the first time, significant global changes in the number of tropical cyclones making landfall as major storms for the period 1982–2020. This significant change is observed based on the intensity at landfall, rather than the lifetime maximum over oceans, which makes this study different from other previous trend analyses, for example, the positive LMI trend^28^, the poleward migration of LMI locations^29^. However, the location of LMI is on average more than 700 km away from the coastline^7^, and it is therefore weakly related to coastal damage directly. A large majority of tropical-cyclone-related damage is caused by the major TC landfalls^9^, so the significant change of landfalls found here is a direct socio-economic threat.

## Methods

### Data

We take the best-track data (LMI ≥ 33 m s^−1^) from the International Best Track Archive for Climate Stewardship (IBTrACS) v04r00^30^. The best-track archive from the Joint Typhoon Warning Center (JTWC) is chosen for the West Pacific (WPAC), North Indian Ocean (NIO), South Indian Ocean (SIO) and South Pacific (SPAC); and the archive from the National Hurricane Center (NHC) covers the East Pacific (EPAC) and North Atlantic (NATL). Considering the best globally consistent best tracks, we choose the best tracks from the US agencies as used by Ref.^10^ whose work we extend here. The original best-track data from JTWC and NHC are recorded every 6 h. In the IBTrACS storm positions and the other measures are then interpolated to 3-h intervals, respectively, using splines and linear interpolation. In this way the IBTrACS data used in our analysis is at 3-h intervals, i.e., 00, 03, 06, 09, 12, 15, 18 and 21 Universal Time Coordinates.

In the period 1982–2020 we have the highest confidence in the quality and completeness of global TC count and intensity observations^31^. A longer period back to 1970 will also be analysed as a sensitivity check. Three criteria are used for the selection of storms:

- the LMI is of at least hurricane-force wind (≥ 33 m s^−1^, i.e., catogary-1),
- storms make landfall, and
- tracks only within 40° N/S are considered to reduce sub-tropical impacts.

The landfall events are selected as labelled in the IBTrACS. The smallest landmass considered in the IBTrACS is 1400 km^2^, equivalent to the area of Kauai, Hawaii. This study is a revisit of Ref.^10^ for global major landfall counts, but at a different land mask resolution. Ref.^10^ used a self-defined land mask with 1/20° global grid spacing that includes more small islands but is not publicly available. In the following analysis we use the standard landfall flag in the IBTrACS so the results can be easily reproduced and extended consistently when more data is available in the future.

Only the landfall with the highest intensity is counted per TC if multiple landfalls occur, and the landfall intensity is the higher of the first land intensity and the last intensity over oceans. Similar results are found if the intensity only over land or oceans is used. The full tracks of landfall TCs over the period 1982–2020 and the locations of major landfalls are shown in Fig. S4.

### Change detection and statistical significance

We examine the count change by Poisson regression^21,32,33^, and intensity and latitude trends by linear trend analysis^28,29^. The statistical significance is defined with 95% confidence intervals (i.e., *p* values ≤ 0.05). The residuals from the linear and Poisson regressions all follow normal distribution (Kolmogorov–Smirnov normality test, *p* > 0.1) and none of them are autocorrelated.

1. Poisson regression
  The Chi-square from the fit of counts to a Poisson distribution is compared to the right-tailed critical Chi-square value with the corresponding degrees of freedom. This analysis suggests that we cannot reject the null hypothesis of a Poisson distribution for any of the count distributions examined here at 95% confidence intervals.
  A generalised linear model is then used for the Poisson regression with the link function $g\left(\mu \right)=ln(\mu )$^15^, where $\mu$ is the Poisson parameter, and ${\mu }_{i}$ represents the annual TC count in year ${x}_{i}$. The Poisson regression model can then be written as
  1 $$ln\left({\mu }_{i}\right)={b}_{0}+{b}_{1}{x}_{i}$$
  the parameters of which are estimated by the maximum likelihood method. Since this model describes an exponential relationship, a doubling time may be defined as
  2 $${T}_{D}=\frac{ln\left(2\right)}{{b}_{1}}.$$
  The confidence intervals (CIs) of the fitted slope ${b}_{1}$ are obtained from its standard error and degrees of freedom. Any autocorrelation is examined with the Durbin–Watson test for the first-order autoregression, or AR(1). If an AR(1) process is detected, the degrees of freedom of the fit is calculated from the effective sample size ${n}^{^{\prime}}\approx n\left(1-{r}_{1}\right)/\left(1+{r}_{1}\right)$, where *n* is the total sample size and *r*_*1*_ is the lag-1 autocorrelation coefficient^15^. The dispersion parameter, estimated by Pearson’s Chi-square statistic divided by the degrees of freedom, is 0.8 for Fig. 1a and 0.9 for Fig. 1b, which suggests that the Poisson regression applied here does not need further adjustment to account for any considerable over or under dispersion.
2. Linear trend analysis

For the annual trend analysis of intensity, the weighted least-squares regression is used, i.e., the annual mean observation is weighted by the annual counts^13^. The CIs of the trend are obtained from the standard error of the linear fit and degrees of freedom. The CIs are adjusted if an AR(1) process is detected, following the same process used in the Poisson regression.

## Supplementary Information

Supplementary Information.

## Data Availability

The tropical cyclone best track data can be downloaded from the National Centers for Environmental Information website (https://www.ncei.noaa.gov/data/international-best-track-archive-for-climate-stewardship-ibtracs/v04r00/access/csv/ibtracs.ALL.list.v04r00.csv).

## Code availability

All codes used to analyse and plot the data are available via https://data.hpc.imperial.ac.uk/resolve/?doi=8701&access=.
