# Supplementary material for: More tropical cyclones are striking coasts with major intensities at landfall
Source: Sci Rep. 2022 Mar 28;12:5236. doi: 10.1038/s41598-022-09287-6 (PMC8960794; doi:10.1038/s41598-022-09287-6)
Supplement: Supplementary file 1 — Supplementary Information. [file 41598_2022_9287_MOESM1_ESM.docx]

**Supplementary Materials for**

More tropical cyclones are striking coasts with major intensities at landfall

S. Wang^1^* and R. Toumi^1^

^1^Department of Physics, Imperial College London, London, SW7 2AZ, UK

*Corresponding author. Email: [shuai.wang@imperial.ac.uk](mailto:shuai.wang@imperial.ac.uk)

**This PDF file includes:**

Figs. S1 to S4

Tables S1


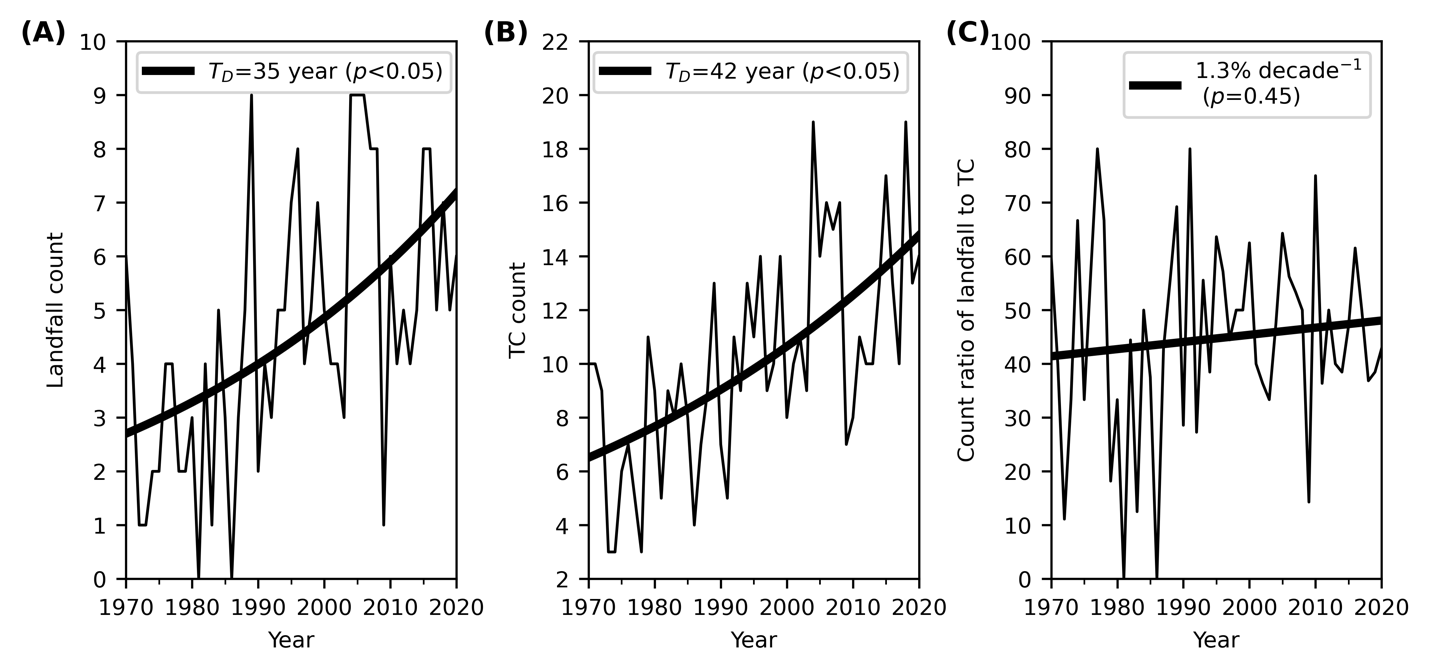


Fig. S1. As in Fig. 1, but with an extended period 1970-2020. For the period of 1970-2010 as used in Weinkle et al. (2012), a consistent R^2^ of 0.09 is found in (a) using a linear regression, but with a trend of 0.7 count per decade (*p*=0.04) and a doubling time T_D_ of 48 year (*p*=0.02). Note that a different landfall calculation from Weinkle et al. (2012) is used here.


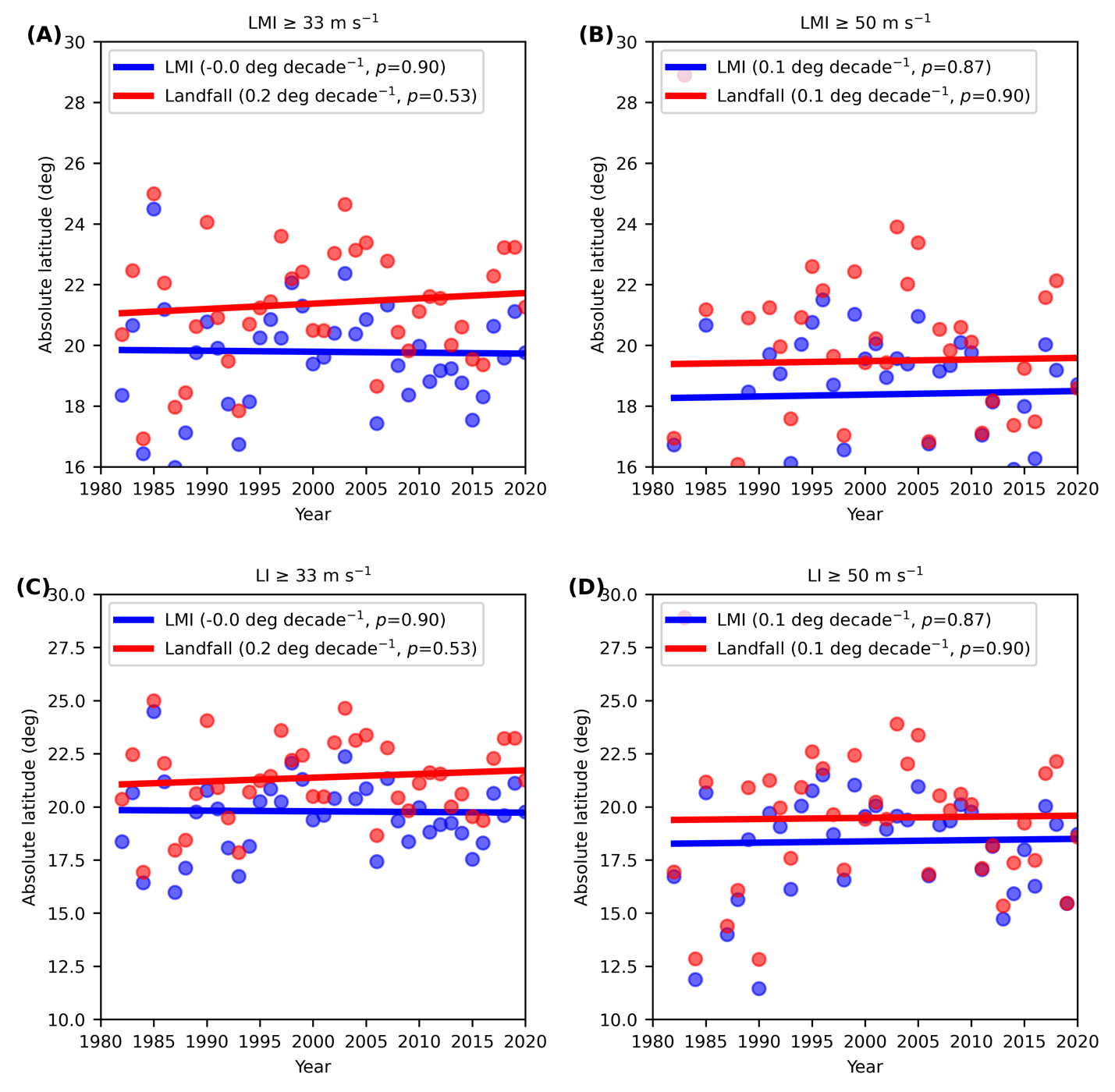


Fig. S2. Global annual mean trend of the absolute latitude of the locations of LMI and landfall for the TCs with (a) LMI ≥ 33 m s^-1^, (b) LMI ≥ 50 m s^-1^, (c) LI ≥ 33 m s^-1^, and (d) LI ≥ 50 m s^-1^.


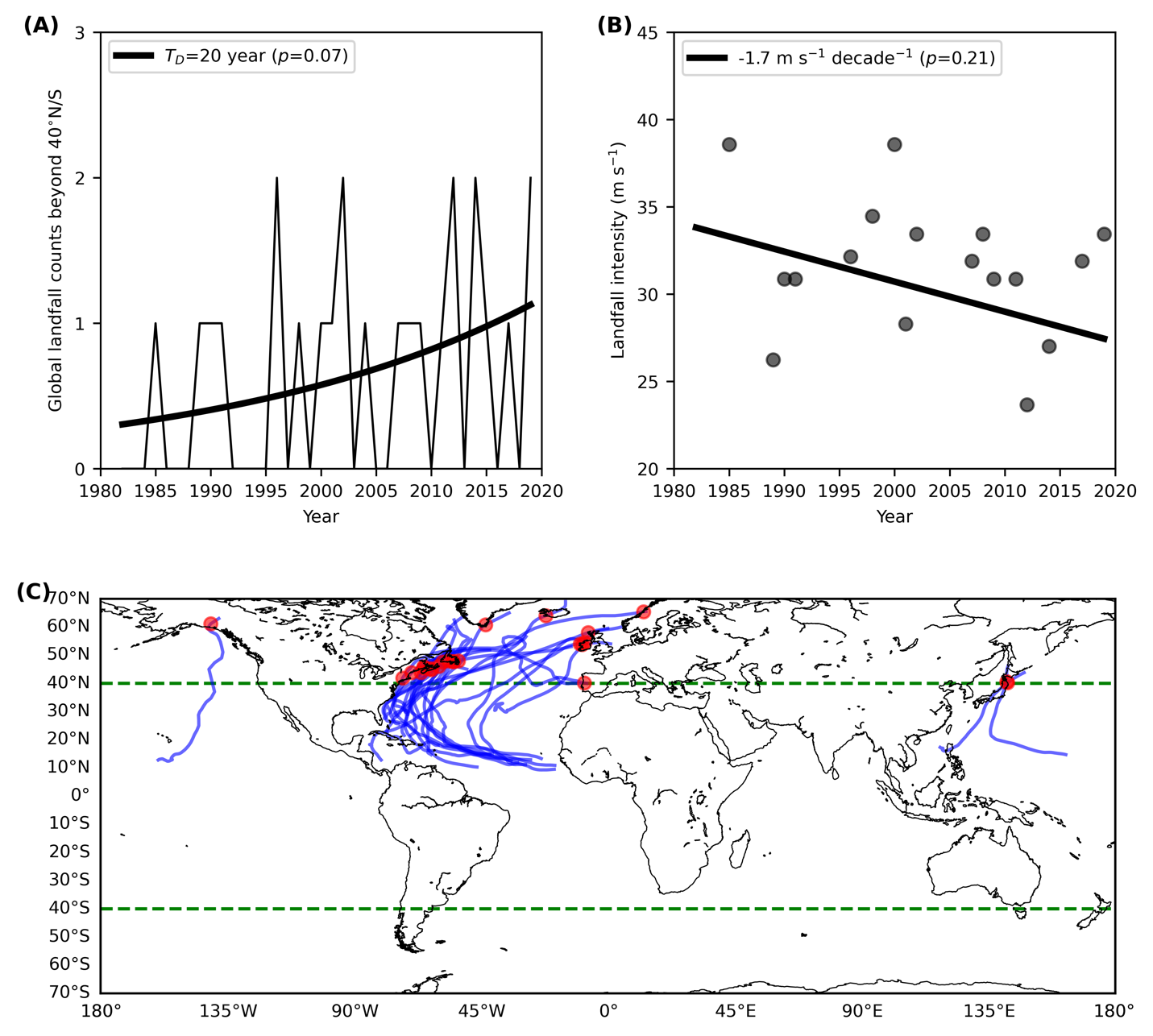


Fig. S3. Long-term change of annual mean (a) landfall count and (b) LI of global TCs with LMI ≥ 33 m s^-1^ and landfall location beyond 40^o^N/S. The thick black line in (a) shows the Poisson regression fit with a fitted slope b_1_ of 0.035 (*p*=0.07). The landfall locations and TC tracks are shown in (c) with red dots and blue lines, respectively. The dashed green lines highlight 40^o^N/S. The map in (c) is generated with the Matplotlib Basemap Toolkit (https://matplotlib.org/basemap/).


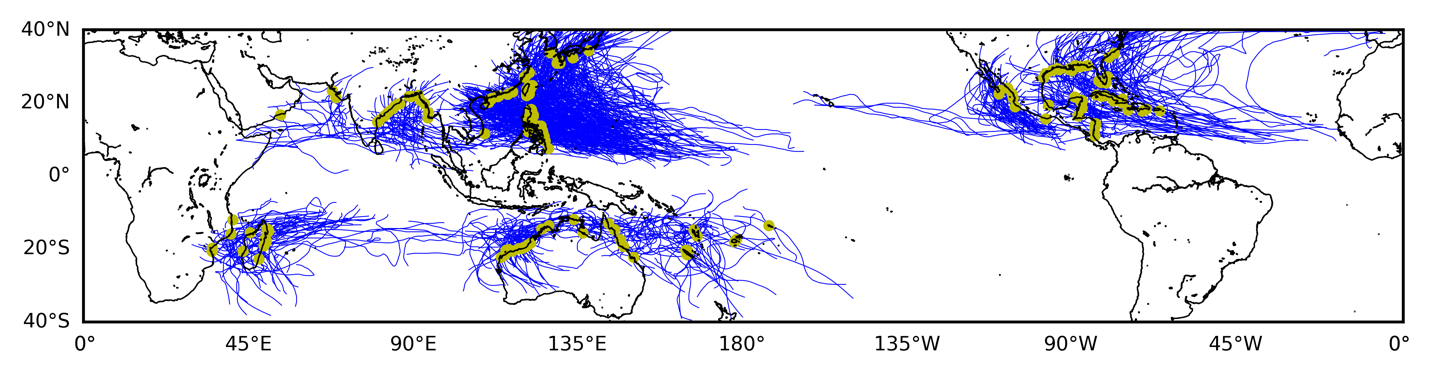


Fig. S4. The full tracks of landfall TCs with the lifetime maximum intensity of at least 33 m s^-1^ (blue lines) over the period 1982-2020. The yellow dots show the locations of landfall as major TCs (LI ≥ 50 m s^-1^). The map is generated with the Matplotlib Basemap Toolkit (https://matplotlib.org/basemap/).

Table S1. Epochal change of LMI and LI between 2020-2001 and 1982-2000 (mean difference±95% confidence intervals) of all the selected landfalling storms with LMI≥33 m s^-1^. The unit is m s^-1^ for LMI and LI. Statistical significance is indicated in bold at 95% confidence intervals (CIs) with bootstrapping. We use the non-parametric bootstrapping to estimate the CIs, considering the distribution of tested variables may not follow the normal distribution. The two tested distributions in the two epochs are resampled 100,000 times with replacement to generate 100,000 pairs of distributions. We then calculate the difference of the means between the resampled distributions in each pair to form a difference distribution that is used to obtain 95% CIs.

|  | Global | WPAC | EPAC | NATL | NIO | SIO | SPAC |
| --- | --- | --- | --- | --- | --- | --- | --- |
| **LI** | 0.9±2.0 | 0.9±2.6 | -0.6±5.8 | 3.5±5.4 | -6.1±7.4 | -0.7±6.0 | **9.0±8.8** |
| **LMI** | **3.7±2.0** | **4.4±2.9** | 5.6±5.9 | 4.0±5.8 | 2.1±7.2 | 0.1±5.4 | 5.6±8.2 |
